# 

**Published:** 1888-04

**Authors:** 


					﻿Vol viil
APRIL, 1888. :
NO. 4/
IFHE
flOMŒOPATHlC pHY^IßlAp,
A Monthly Journal of Homoeopathic Materia Medica
and Clinical Medicine.
“ He is a freeman whom truth makes free, and all are slaves beside.”
“Seek the Truth: come whence it may, cost what it will.”	■ ■ >
EDITED AND PUBLISHED BY
Edmund j. lee, M. d.,
AND
WALTER M. JAMES, Ni. D.
PHILADELPHIA :
No. 11S3 SPRUCE STREET.
LONDON:
ALFRED HEATH & CO., Sole Agents fob Great Britain,
114 Ebury Street, Eatou Square.
^¡'Yearly Subscription, $2.SO. invariably in advance. Single numbers, 25 cts.
Entered at the Philadelphia Poet-Office aa Second- Claas Mail Matter.
				

